# Animal Cruelty and Neglect: Prevalence and Community Actions in Victoria, Australia

**DOI:** 10.3390/ani9121121

**Published:** 2019-12-11

**Authors:** Carmen Glanville, Jennifer Ford, Grahame Coleman

**Affiliations:** 1 Faculty of Veterinary and Agricultural Sciences, the University of Melbourne, Animal Welfare Science Centre, North Melbourne, VIC 3051, Australia; grahame.coleman@unimelb.edu.au; 2 Royal Society for the Prevention of Cruelty to Animals (RSPCA) Victoria, Burwood East, VIC 3151, Australia

**Keywords:** animal mistreatment, cruelty, neglect, prevention, prevalence, reporting, animal welfare, attitudes

## Abstract

**Simple Summary:**

Preventing animal cruelty and neglect is the goal of animal protection. But it is hard to effectively address a problem without a good understanding of its prevalence and nature. While 55,000–60,000 reports of mistreatment are made to the Royal Society for the Prevention of Cruelty to Animals (RSPCA) in Australia each year, we do not know how well these data reflect what is actually happening in the community. After all, these data are reliant on people *reporting* what they see and therefore, probably only represent a fraction of what is actually occurring. To better understand this problem, we conducted the first extensive community survey to find out what people had seen in their communities and what they did about it. We found that animal mistreatment was (1) common, with 25.7% of people surveyed witnessing mistreatment, (2) mostly neglect with perceived underweight animals being the most common issue and (3) underreported to authorities with only 9% of witnesses reporting to RSPCA Victoria. While sobering, these findings are the first step to developing and resourcing well-informed strategies to prevent the mistreatment of animals.

**Abstract:**

While animal mistreatment is common worldwide, its true scale is largely unknown. Currently, organisations rely on community reporting (case data) and trends found therein to inform prevention activities. To investigate the prevalence, types, and responses to animal mistreatment in Victoria, we conducted a representative telephone survey (*n* = 1801) across six Local Government Areas (LGAs); three with high numbers of RSPCA reported cases and three demographically similar areas with low numbers of such cases. Overall, 25.7% of people surveyed had witnessed at least one incident of mistreatment in the last 12 months, with those relating to neglect or poor management predominating. No differences in prevalence were found between LGAs when socio-economic index and local government comparator group were controlled for. However, participants in regional cities recalled witnessing more separate incidents than those in metropolitan or interface areas. Actions taken after witnessing mistreatment were varied, yet many participants did nothing (27%) and only 9% reported to RSPCA Victoria. Attitudes to reporting were positive but did not predict reporting behaviour. Together, these results demonstrate that case data are not reliable indicators of the true prevalence of animal mistreatment; it is common and grossly underreported, highlighting the need for effective, evidence-based prevention programs.

## 1. Introduction

Animal mistreatment is a complex issue that affects countless animals worldwide [1]. Differences exist in the literature and the common vernacular regarding the use and meaning of terms such as cruelty, neglect, abuse and maltreatment. As such, here we will use the term ‘animal mistreatment’ to refer to all instances of significant animal suffering caused by a human, including both neglect and cruelty, regardless of intent. In Australia, around 55,000–60,000 reports of animal mistreatment are made each year to the RSPCA [2], approximately 11,000 of which are made in the state of Victoria [3]. While this is a substantial number, it is likely that, similar to other related social issues such as domestic violence and child abuse [4,5,6], these reports are just the ‘tip of the iceberg’ [7]. Taking the lead from more developed fields of prevention and intervention such as interpersonal violence and public health, understanding the extent of such an issue is the first crucial step in developing informed intervention strategies [8]. Previous research has investigated the prevalence of overt animal cruelty in non-representative samples of various subpopulations (e.g., adolescents [9], domestic violence perpetrators [10], and prison inmates [11]). Additionally, one study examined the prevalence of self-reported intentional animal abuse in two cities in Russia and Ukraine [12]. However, to date, there has been no empirical investigation of the prevalence of animal mistreatment (including both neglect and cruelty) in the general community and it is unknown how accurate a reflection official case data (reports made by the community) are of what is actually occurring in the community. Consequently, it is likely that the true scale of the problem has been largely overlooked. It is crucial to understand this for informed and effective policy and resourcing decisions to be made.

Using the only information available to them, animal welfare organisations and researchers have taken to analysing case data and even more limiting, prosecution records, for various trends [13,14,15]. Analysis of the Victorian (Australia) data has found that the number of cases differ significantly between Local Government Areas (LGAs). That is, some LGAs have consistently high numbers of cases, while others have low numbers of cases. These results are often communicated in the mainstream media, identifying ‘cruelty hot spots’ [16,17,18,19,20,21,22]. However, it is also found that the majority of complaints relate to issues of neglect rather than intentional cruelty. The observed differences between LGAs have also prompted interest in region specific intervention programs where there are high numbers of cases [23]. However, it is unknown if these differences in the number of cases between LGAs represent a true difference in prevalence of animal mistreatment, or merely differences in reporting. Indeed, many factors may influence reporting behaviour and an alternate explanation of these trends is that people in areas with high numbers of cases actually care *more* about animals and are therefore more likely to report when they see something wrong.

Consequently, in order to understand how case data trends relate to the actual prevalence of mistreatment, it is also important to understand how and why members of the community respond to witnessing instances of mistreatment; what do they do and why do they do it? Of particular interest is why people choose to report (or not) to authorities. While factors influencing reporting have been examined in other criminal situations [24], only one study that we are aware of has investigated this with regard to animal mistreatment. Taylor and Signal [25] investigated the relationships between several, mainly demographic, factors and people’s propensity to report mistreatment. They found that individuals who identified as female, those working in ‘white collar jobs’, and those with an awareness of the link between animal cruelty and family violence, had a greater propensity to report mistreatment. While these are interesting findings, only one of these factors is open to change (awareness of the link between animal cruelty and family violence) and it would be beneficial for prevention initiatives to understand more about the attitudinal factors that influence reporting.

Therefore, the aim of this study was to gain a more objective understanding of the prevalence and types of animal mistreatment in Victoria, as well as how the community responds to witnessing mistreatment and why. Specifically, we aimed to determine how areas with high numbers of RSPCA Victoria cases differed with respect to these factors (prevalence, types, actions, and attitudes) to similar areas with low numbers of cases. Not only will this information prove valuable for decision making and planning of prevention activities in Victoria, it will also serve as an indicator of the accuracy of case data and trends for organisations worldwide.

## 2. Materials and Methods 

### 2.1. Human Ethics Approval 

This project was conducted in accordance with the National Statement on Ethical Conduct in Human Research (2007) guidelines and regulations. Ethics approval for both the formative focus groups (ID: 1853397) and survey (ID: 1954263) was granted by the University of Melbourne Veterinary and Agricultural Sciences Human Ethics Advisory Group. All participants provided informed consent prior to partaking and were given the opportunity to withdraw their data upon completion of the task.

### 2.2. Focus Groups

Four focus groups, consisting of between six and twelve participants, were conducted in February 2019. The aim of the focus groups was to elicit common attitudes and beliefs from the general community to inform item development for the subsequent Computer Assisted Telephone Interview (CATI) questionnaire. The sessions were conducted in person at community spaces (library and a neighbourhood house) in the City of Latrobe region. The City of Latrobe region was used because a separate project, for which it was the focal site, provided the opportunity to conduct this work. Adult members of the general community were recruited through advertisements in social media groups (buy/swap/sell groups and community noticeboard groups) and provided with a financial incentive ($60) to attract a range of views. The final sample consisted of 37 individuals, 26 female, 10 male, and 1 gender non-binary, aged from 18 to 65 years. No recruits were refused participation. Key themes discussed included attitudes towards animals, what constitutes mistreatment, and perceived prevalence of mistreatment. Common themes and attitudes were identified for inclusion in the questionnaire.

### 2.3. Survey Questionnaire 

The questionnaire was designed to investigate (1) the prevalence of mistreatment, (2) the actions taken after witnessing mistreatment and, (3) people’s attitudes towards reporting mistreatment. Demographic questions (age, education, country of birth, income bracket) were also included.

To estimate the prevalence of animal mistreatment, we investigated the number of incidents of mistreatment participants could recall witnessing in the past 12 months. However, animal mistreatment is a subjective topic; what one person considers to be mistreatment, another may not. As such, instead of simply asking whether a person had witnessed ‘mistreatment’, participants were asked ‘In the last 12 months have you seen in your neighbourhood:’ and then explicit descriptions of ten common types of animal mistreatment (as identified from RSPCA Victoria cases) were read out (see Table 1). If they answered yes to any of these, they were asked on how many separate occasions (i.e., involving different animals) they had seen this and the numeric value was recorded. These descriptions were based on commonly used descriptors or guidelines used by RSPCA employees when determining whether a situation likely involves mistreatment.

For each type of mistreatment that a participant had witnessed, they were then asked what they did, or most recently did if there were multiple occasions, after witnessing it. Participants selected one option for each type of mistreatment they had witnessed from the following list: 1. Made a report to RSPCA, 2. Made a report to the local council, 3. Made a report to a government department or other statutory authority, 4. Made a report to Police, 5. Discussed your concern with a professional, e.g., vet, animal welfare worker, 6. Sought advice from a family member or friend, 7. Other, (please specify), 8. Nothing. The response options were randomised in order to prevent response bias.

For each type of mistreatment witnessed that the participant had not acted upon (responded ‘nothing’ to the previous question), they were asked to describe in a few words why they had not acted. Their responses were then categorised by the telephone interviewer to pre-programmed responses: 1. I was unsure whether mistreatment was actually taking place, 2. I was not sure whether it was against the law, 3. I didn’t know what the right thing to do was, 4. I didn’t want to get involved, 5. I didn’t know who to contact, 6. I didn’t think it was any of my business, 7. I thought I might make the situation worse for the animal, 8. Someone else told me not to, 9. I was concerned about the person finding out it was me, 10. I was concerned about retaliation or backlash from the person involved, 11. I didn’t think the authorities would help, 12. I was worried the animal would be euthanized, 13. I didn’t have time, 14. Other (Specify), 15. There were no such situations (I always did something). After data collection was complete, five additional categories that were frequently noted in the ‘Other (Specify)’ response were added and back-coded: 16. I thought the situation was improving, 17. Stray or feral animal, 18. Witnessed in passing and couldn’t intervene, 19. Someone else was taking action, 21. Wildlife.

Participants were also asked a series of attitudinal questions related to reporting animal mistreatment. These items were modelled off the Theory of Planned Behaviour (TPB), one of the most widely used models of human behaviour [26]. The TPB identifies behaviour as an outcome of three attitudinal elements: (1) attitudes towards the behaviour itself, (2) subjective norms, and (3) perceived behavioural control [27]. While attitudes are difficult to measure directly, they can be inferred by responses to belief statements related to these three elements; (1) beliefs about the behaviour itself and its outcomes, (2) normative beliefs about how significant others would expect the individual to behave, and (3) beliefs about the degree of control the individual has over performing the behaviour [27]. Relevant beliefs were identified through the focus groups and in consultation with industry experts. Two additional belief statements that did not clearly fall into these categories were also incorporated as they were commonly expressed by focus group participants. Attitudinal items were scored on five-point Likert-type scales (Table 2) and randomised in order.

### 2.4. Local Government Area Selection 

As one of the key aims of the study was to determine the accuracy of case data with regard to observed trends between LGAs, select LGAs were chosen for sampling as opposed to a state-wide representative survey. A representative survey of Victoria would not allow for comparison of LGAs or identification of trends, because, unless the sample size was very large, the number of participants from each area would be small and provide unreliable estimates. Six LGAs were chosen for inclusion in the survey; three with high numbers of RSPCA reported cases and three with low numbers of cases (Table 3). To do this, all 79 Victorian LGAs were ranked based on the raw numbers of reported cases and per capita cases over the preceding 3 years (2015–2018). The ten highest ranked (high numbers of cases) and ten lowest ranked (low number of cases) LGAs were considered for selection. Of the ten highest ranked (HR) LGAs, a regional city (City of Latrobe) was selected, being the highest ranked, along with an inner-city region (City of Melbourne) and an interface (peri-urban) region (Yarra Ranges Shire), to gain a better representation of the various region types that exist within Victoria. These three HR LGAs were then paired with similar low ranked (LR) regions based on Local Government Comparator Groups (standardised categories of councils developed to enable meaningful comparison of data and services between Victorian councils [28]), population, and Social Economic Index for Area (SEIFA) scores. The City of Latrobe was matched with Mildura Rural City and City of Melbourne with City of Stonnington. No interface councils appeared in the ten lowest ranked LGAs. Consequently, the interface councils with the lowest rank were considered, despite them not appearing in the bottom ten. While Nillumbik Shire had the lowest rank of the interface councils, Wyndham City was selected as it better matched the demographics (population and SEIFA ranking) of the chosen HR LGA (Yarra Ranges). This pairing of HR and LR LGAs allowed for more meaningful interpretation of results by limiting the number of variables between comparators.

### 2.5. Questionnaire Delivery and Sampling

Questionnaire delivery was contracted to the Social Research Centre, a data collection subsidiary of the Australian National University specialising in social and health research. The questionnaire was delivered as a Computer Assisted Telephone Interview (CATI) using random digit dialing. Telephone number lists were acquired by the Social Research Centre from a commercial provider with a mixture of 80% mobile numbers and 20% landlines. For mobile numbers, a pre-notification SMS message was sent prior to phoning to provide the opportunity to opt-out and improve the likelihood people would answer the phone.

To facilitate a more representative sample, in each call the operator requested to speak to the person living in the household aged 18 years or over who was to have the next birthday. Population representative quotas were set for gender and age.

Data collection spanned four weeks from 29 April to 27 May 2019. The AAPOR Response Rate 3 [30] was 9.4% and the overall cooperation rate was 19.5%, with significant variation between landline (31.8%) and mobile (18.1%) frames.

### 2.6. Data Analysis

All data analyses were conducted using IBM SPSS Statistics for Windows, version 25 (IBM Corp., Armonk, N.Y., USA), except for the Rodgerian analyses which were conducted in the Simple Powerful Statistics application [31]. All variables were screened using descriptive analyses. Extreme outliers (z > 3.29) were removed for the variables related to the number of separate incidents witnessed; each of these values was greater than 300 and it was likely the participant had misunderstood the question. While the distributions of responses were often skewed, given the large sample size (*n* = 1801), normality of sampling distribution was assumed in most cases.

Rodger’s method of decision-based contrasts was used to compare between LGAs both the proportion of people who had witnessed mistreatment and the proportion who had reported to RSPCA Victoria. Rodger’s method was utilised as it is the most powerful post-hoc statistical procedure for detecting differences in proportions between groups owing to its use of decision-based type 1 error rates as opposed to experiment-wise error rates [32,33]. Two-way factorial Analysis of Variance (ANOVA) was used to compare the mean number of incidents witnessed between HR/LR LGAs and the different region types (regional, interface, metro).

Correlations between attitudinal variables were low (<0.3) and hence, not suitable for dimension reduction techniques [34] (p. 685). As such, all attitude items were used in the analyses reported here. The relationship between attitudes towards reporting and reporting behaviour was first examined with biserial correlations. Correlations were only run on cases where the participant had witnessed mistreatment and had the opportunity to report (*n* = 462). The correlations between attitude items and reporting behaviour were weak and while logistic regression and discriminant function analysis were trialed, they yielded poor results accordingly. As such, these analyses were not appropriate and have not been reported. The relationship between reporting and other demographic variables was examined using chi-square tests.

## 3. Results

### 3.1. Participant Demographics

A total of 1801 individuals were surveyed with 300 respondents each from Latrobe, Yarra Ranges, Melbourne, Stonnington, and Wyndham, and 301 from Mildura. Respondents ranged in age from 18 to 93 (*M* = 49.32, *SD* = 16.86) with 45.6% identifying as male, 53.7% female, 0.2% as other gender, and 0.4% undisclosed (0.1% discrepancy due to rounding). The majority of respondents (64.1%) had animals in their households with dogs (46.4%) and cats (27.2%) being the most common. Most respondents (70.0%) had completed year 12 or equivalent and 79.4% had completed further qualifications (including trade certificates).

### 3.2. Prevalence of Animal Mistreatment

Across the whole sample (*n* = 1801), 462 respondents (25.7%) had witnessed *at least one* incident of animal mistreatment in the past 12 months. The proportion of people who had witnessed at least one incident of mistreatment did not differ between paired HR/LR LGAs (Table 4).

Overall, the mean number of *separate incidents* witnessed was 10.8 (*SD* = 42.06) with a median of 2. Again, the mean number of separate incidents witnessed did not differ between paired HR/LR LGAs, *F_1,444_* = 0.217, *p* = 0.642. However, the average number of incidents witnessed was significantly higher in the regional cities (*M* = 17.16, *SD* = 57.60, *n* = 183), when compared to the interface (*M* = 5.85, *SD* = 21.52, *n* = 127) and metro regions (*M* = 7.12, *SD* = 29.34, *n* = 140), *F_2_*_,444_ = 3.554, *p* = 0.029. While Levene’s test of homogeneity of variance was violated for this factorial Analysis of Variance (ANOVA) (*p* < 0.001), the sample sizes were similar between groups (ratio between largest group and smallest group <1.5) and hence, robust to violation [35] (p. 361). Additionally, as the group with the larger sample size had the larger variance, this would result in a loss of statistical power, and yet a significant difference was still detected. No interaction effect was found between HR/LR and LGA type.

### 3.3. Types of Animal Mistreatment Witnessed

**Figure 1:**
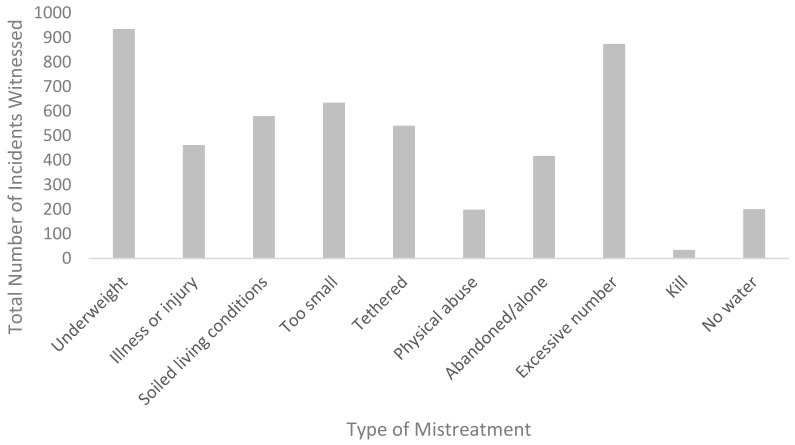
Underweight animals and excessive number of animals were the most common types of mistreatment witnessed across the sample.

As demonstrated in Figure 1, the most commonly witnessed form of mistreatment overall was underweight animals (935 separate incidents), followed by excessive numbers of animals such that the owner could not care for them appropriately (874 incidents).

**Figure 2:**
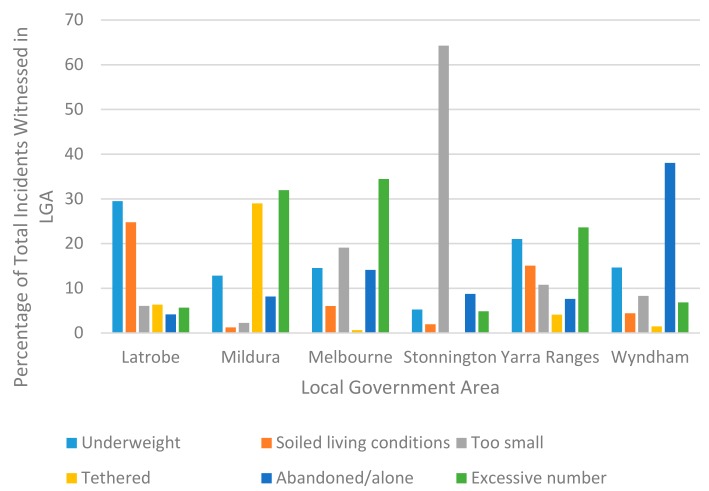
Most common types of mistreatment witnessed differed between Local Government Areas (mistreatment types with low frequency omitted for ease of visualisation).

However, the most common forms of mistreatment witnessed differed between the LGAs (Figure 2).

### 3.4. Actions Associated with Witnessing Animal Mistreatment

**Figure 3:**
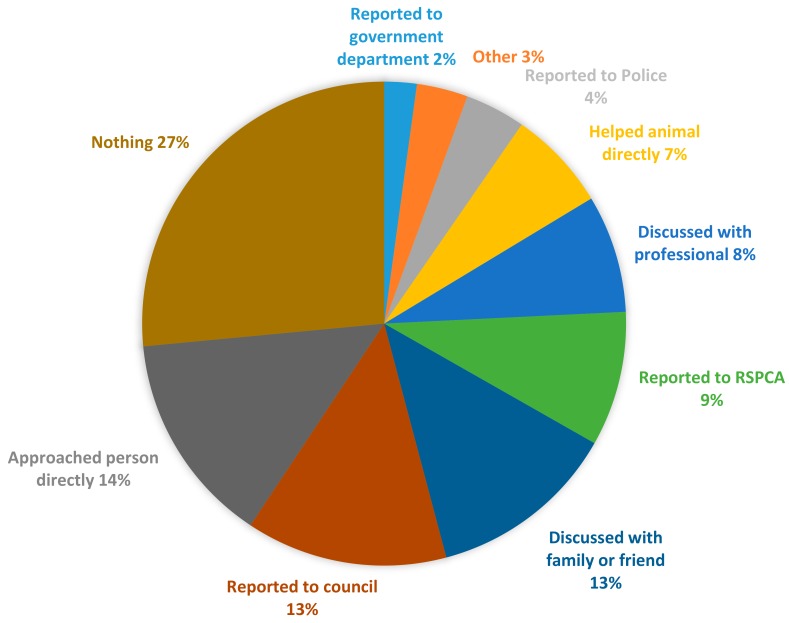
Actions taken after witnessing animal mistreatment were varied. Actions expressed as percentages of the total number of responses.

For each type of mistreatment a participant had witnessed, they were asked what they did in response, or what they most recently did if there were multiple incidents of the same mistreatment type. The sum of action responses for each LGA and the sample as a whole is provided in Table 5 with a graphical representation of their proportions provided in Figure 3. While 462 participants had witnessed mistreatment, many had witnessed multiple different types, hence the total number of recorded responses was 967.

When all actions were summed together, participants most commonly took some form of action (73% of all actions recorded). However, reporting to the various types of authorities (RSPCA Victoria, council, government, police) was relatively low with only 9% reporting to the primary investigatory body for animal mistreatment, RSPCA Victoria. The single most frequent response overall was to do nothing (27% of all recorded actions). This was consistent across all LGAs individually except Wyndham, where the most common response was to discuss the situation with friends or family.

While no action was the most common response for five out of the six LGAs, other responses to mistreatment differed between HR/LR LGA pairs. Specifically, reporting to RSPCA Victoria differed significantly in the regional city and metropolitan pairs (Table 6). People in the HR LGAs (Latrobe and Melbourne) reported to RSPCA Victoria more often (for more types of mistreatment) than those in their paired LR regions (Mildura and Stonnington). However, there was no statistically significant difference for the interface pair.

Reponses to witnessing animal mistreatment also varied between different types of mistreatment. No action was the most common response for eight of the ten types of mistreatment. However, when an animal was killed, the most common response was to report it to the police (38%) or council (23.1%) and when an animal was without water, participants most commonly either helped the animal directly (28.2%) or reported to the RSPCA Victoria (28.2%).

### 3.5. Reasons for Inaction

**Figure 4:**
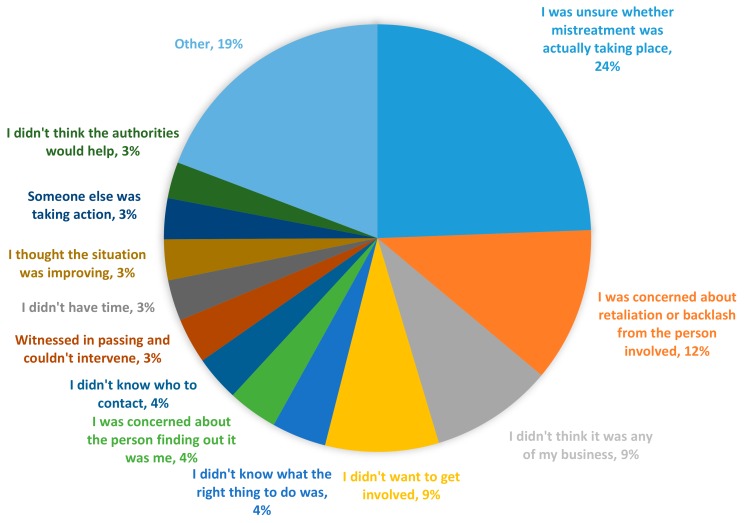
Reasons given for not taking action after witnessing animal mistreatment expressed as a percentage of total responses (*n* = 291).

The most common reason given for not taking action after witnessing animal mistreatment was being unsure that mistreatment was actually taking place (24%) (Figure 4). However, when asked how confident they would be in recognising the signs of mistreatment, only 9% of these same people responded that they were ‘not very confident’, with 28.4% being ‘fully confident’ and 14.9% ‘mostly confident’. The next most common reasons given for inaction were fear of retaliation (12%), thinking it was ‘not any of my business’ (9%), or not wanting to get involved (9%). The ‘other’ section includes both actions that did not fall into any of the pre-programmed responses (e.g., praying, ongoing monitoring of situation, informing supervisors) and pre-programmed responses that had less than a 3% response rate for ease of visualisation.

### 3.6. Attitudes towards Reporting Mistreatment

Generally, attitudes towards reporting animal mistreatment were positive. Across the whole sample (*n* = 1801), most people considered that reporting mistreatment would help the animal (88.3%) and that it is the right thing to do (98.8%). However, there were still 125 people (11.7%) who thought that it would not help the animal.

Biserial correlations between the attitude items and whether a person reported mistreatment were weak (Table 7). While four items were statistically significant, the correlation coefficients (r) were small (<0.16) and likely to be of little practical significance. This conclusion was supported by the poor results produced by more detailed statistical analyses (logistic regression and discriminant function analysis not reported here).

### 3.7. Associations between Demographic Variables and Reporting 

As shown in Table 8, the only demographic variables that indicated a significant relationship with reporting behaviour were the participant’s LGA and LGA type (i.e., regional vs. metro vs. interface).

## 4. Discussion

This study aimed to gain a more objective understanding of animal mistreatment in Victoria and how this relates to previously relied on RSPCA Victoria case data. To do so, it is important to examine both the prevalence of witnessing mistreatment and the actions taken by witnesses. Collectively, our results suggest that there is a much larger problem of animal mistreatment in Victoria than previously considered and that official case data trends are not an accurate reflection of the prevalence of mistreatment within individual LGAs.

Overall, witnessing animal mistreatment was common with 25.7% of participants having witnessed at least one incident in the past year, often many more. As we only sampled six of the 79 LGAs in Victoria, generalisations are limited. However, given that the three main region types were represented (regional, interface, metro), it is reasonable to suggest that the average proportion of people who had witnessed mistreatment would be of a similar magnitude across the state. Of particular note is that only 9% of the people witnessing mistreatment reported what they had seen to RSPCA Victoria, the main regulatory body in Victoria for such reports. If this figure is consistent across the state, then it follows that the 11,000 reports made to RSPCA Victoria each year may only represent 9% of the true problem.

The differences in prevalence between LGAs found in RSPCA Victoria case data are likely more reflective of reporting propensity than of true differences in prevalence. When LGAs with high numbers of RSPCA cases (HR) and low numbers of RSPCA cases (LR) were matched based on socioeconomic index, population, and local government comparator groups, they did not differ in either the number of people who had witnessed at least one incident of mistreatment or the average number of separate incidents witnessed. This interpretation is also supported by the differences between the stated actions of participants in the regional and metro HR and LR LGAs; those in HR areas indicated that they reported to RSPCA Victoria more than those in LR areas. Not only do these results indicate that so-called ‘hot spots’ are falsely labelled, but also that a closer examination of areas with low reports may be required as they are likely to have a similar prevalence of mistreatment, but given their low reporting, the animals are not receiving the assistance they need.

However, it is important to note that this trend was not maintained with the interface pair; no statistically significant difference was found between the reporting behaviour of people in the LR Wyndham and HR Yarra Ranges. While it is unclear exactly why this may be, it is possible that this is because the interface LR LGA selected for the study (Wyndham) was not actually in the lowest 10 ranked LGAs (refer to methods for explanation). However, this provides a potentially interesting insight; being the only LGA surveyed that was not at the extreme ends of the case data rankings and given that it demonstrated the lowest prevalence rates combined with a similar propensity to report mistreatment as the HR LGAs, it suggests that those LGAs in the middle of the rankings may actually have the lowest true prevalence. Further investigation of other middle ranked LGAs would be required to elucidate this theory.

While the majority of participants who had witnessed mistreatment took some form of action, our results suggest that there may be uncertainty or conflicting motivations with regards to what the most appropriate course of action is. This is evidenced by the range of different actions taken and that reporting to the primary enforcement body (RSPCA Victoria) was low. That the single most common response to witnessing mistreatment was no action (27%) is also concerning. However, it is consistent with what is known as the ‘bystander effect’ which is well documented in other areas such as sexual harassment [36], sexual assault [37], and shoplifting [38]. These findings raise significant questions about what influences whether and how a witness will act, specifically whether they will report. In this study, reporting behaviour was difficult to predict. Unlike Taylor and Signal [25], we found no relationship between demographic variables and reporting behaviour (other than location). While there are well documented relationships between demographic factors such as age and gender and attitudes towards animals [39,40,41], given our findings that attitudes themselves had poor relationships with reporting, this is logical. Indeed, most people surveyed had relatively positive attitudes towards reporting mistreatment; they thought it was the right thing to do and that it would help the animal, yet still did not report. Reasons given for not acting in general were largely related to uncertainty (not being sure that mistreatment was taking place) and concern for personal consequences (fear of retaliation or not wanting to get involved). However, the uncertainty of witnesses was likely not a result of not knowing what mistreatment looks like, but rather a result of other contextual factors; most people who said that they did not act because they were unsure mistreatment was taking place, stated in the attitudinal questions that they were confident they could recognise mistreatment. Additionally, given the level of concern for personal consequences, the relationship with the offender and the characteristics of the offender are also likely to be important considerations in the decision to report [38]. Incidents involving friends, family, neighbours, or individuals who are volatile, would likely reduce an individual’s propensity to report. Additionally, in situations where the witness is the only person likely to have seen the incident, making it easy to identify who made the report, it is likely that this would impact reporting behaviour due to potential negative consequences. Another influential factor that has been identified in other fields is the seriousness of the offence [24]. This was reflected to some degree by the differences in actions given the type of mistreatment occurring. In more serious situations, such as when an animal was killed, participants were most likely to report to police, whereas in simple situations like an animal with no water, they helped the animal directly. The only factor that was found to have a significant association with reporting to RSPCA Victoria was where the participant lived, i.e., their LGA. Hence, factors related to the social environment and potentially service provisions in individual areas, like the visibility or availability of RSPCA in that area may be influential. Mistrust or perceived powerlessness of authorities is a significant factor in other fields [42], though only 3% of people in this study identified this as their reason for not acting. Consistent with other bystander intervention work [36,43], these findings indicate that responding to mistreatment, specifically reporting, is a complex behaviour with a range of external or contextual factors not accounted for here playing a significant role.

While individual LGAs with similar socio-economic contexts did not differ in prevalence, significant differences were found between region types. Participants in the regional cities surveyed recalled witnessing approximately 2.5 and 3 times more separate incidents than participants in the metro and interface regions respectively. This is not reflected in the RSPCA Victoria case data and without further information we cannot say definitively why this would occur. However, one possible explanation is that both regional cities surveyed were areas of significant social and economic disadvantage, ranked 3rd and 5th most disadvantaged in the state [29]. Financial difficulties and low education rates have previously been identified as predictors of animal neglect [14,44]. As such, support and educational strategies as opposed to punitive approaches may be most appropriate in these areas.

Neglect-based issues, failing to provide for the animal’s basic needs, were the most common types of mistreatment witnessed. This is consistent with trends in RSPCA cases in Victoria [45] as well as those in Queensland [13] and America [14,15,46]. The differences between LGAs in the most common types of mistreatment likely reflect the different lifestyles and pressures experienced in those areas. For example, the most common issue witnessed in Stonnington was animals being kept in areas they could not move around freely in. This is understandable given that Stonnington is a high density metropolitan area with large numbers of apartment blocks. In contrast, the most common issue in Latrobe was underweight animals, which likely reflects the economic difficulties faced in this area. Consequently, when considering targeted prevention strategies, it would be beneficial to consider these contextual challenges in order to tailor programs to the needs of that community.

As a whole, these findings raise significant questions about prevention messaging and the current trend of mass communications calling out areas with high numbers of reports. In multiple states of Australia, so-called ‘cruelty hotspots’ are highlighted in the mainstream media with pejorative headlines like ‘Caboolture named and shamed over cruelty’ [16,17,47]. Not only is this likely to be inaccurate given the present research, such descriptions may have unintended negative consequences. Studies of mass communications and interventions in a range of other fields suggest that such consequences could include ‘boomerang effects’, social norming, or desensitisation leading to apathy [48,49,50,51,52], all of which work against prevention aims. In addition, the use of the term ‘cruelty’ is likely to have certain connotations with the public that are not reflective of the majority neglect cases. One particular concern is the potential for these messages to marginalise individuals who are at-risk of neglecting animals as a result of their social and economic circumstances. In these instances, the most desirable behaviour is for them to seek help. Yet, by shaming or isolating individuals this may result in a reluctance to do so for fear of judgement or persecution [51].

As the first study of its kind, we recognise its limitations. Firstly, in the absence of being able to either ask animal owners about their experiences (i.e., whether they mistreat their animals) or directly observe the treatment of animals, we must rely on mistreatment being witnessed by others. Given that mistreatment largely occurs in the home environment, there are significant barriers to this and likely much that goes unnoticed. As a participant in one of our preliminary focus groups noted, ‘It’s the ones you don’t see that you need to worry about’. Additionally, the accuracy with which an individual can recount the number of separate occasions they had witnessed mistreatment in the last year is questionable. Hence, we did not use those particular figures to make generalisations about mistreatment, they were simply used to compare averages between LGAs. In doing so, we assume that any error of measurement would be consistent across regions; we have no reason to believe that people in one region would have a more accurate recollection than those in another. As the questionnaire was presented in English and translation into other languages was beyond the scope of this study, there is potential for sampling bias. However, of the total number of individuals contacted to participate, only 0.8% were excluded as they did not speak English. With the questionnaire delivered by CATI and the participant interacting by phone with an interviewer, there is also potential for social desirability bias, particularly with regards to questions about attitudes and actions taken. However, other methods of survey delivery that would reduce this (e.g., online or mail) would not have been suitable; online surveys cannot facilitate random sampling (no sampling frame) and mail out surveys have low and slow response rates. Finally, as with any survey of this nature, there is the potential for those who are more concerned about animals and animal welfare to be more likely to agree to participate. This has the potential to bias the sample, but short of studying those who refuse to participate and comparing them to our sample, it is impossible to quantify this.

## 5. Conclusions

In the study of animal mistreatment, our ultimate goal is to prevent it from happening. In order to facilitate well-informed decisions about policies, resourcing, and prevention programs, authorities require accurate information about the problem. The results of the present study are the first step towards achieving this, demonstrating that animal mistreatment is common, underreported, and mostly neglect. Henceforth, ongoing monitoring would be beneficial to track trends over time and evaluate the impact of prevention initiatives. Additionally, given the low reporting rates, strategies to increase reporting would serve to make the information readily available to authorities (case data) more reflective of the problem. However, care needs to be taken in the development of such interventions as unintended consequences can arise. We would recommend an approach that does not focus on the prevalence of mistreatment or case data, but rather destigmatises reporting and frames it as a way to seek help for others who may be struggling. Any such strategies would need to be tested for their effectiveness and consequences. Overarchingly, this study highlights the need for investment in effective, evidence-based prevention programs to address the substantial amount of animal suffering occurring in our communities each day.

## Figures and Tables

**Table 1:** Types of mistreatment included and descriptions provided in the questionnaire.

Mistreatment Type	Description
Underweight	An animal that was underweight, such that you could see its ribs or hip bones
Insufficient treatment	An animal with an obvious illness injury or other physical health related issue, that you believe was not receiving veterinary treatment
Unhygienic living conditions	An animal being kept in an area that was heavily soiled with poo/faeces
Confined	An animal that was often kept in an area that was too small for it to move around freely
Tethered	An animal that was tied up for more than 22 h a day
Physical abuse	A person intentionally hit, kick, or otherwise physically harm an animal
Unattended	A dog or cat left alone for days at a time with nobody attending to it
Excessive number	A person with too many animals to look after them all properly
Kill	A person intentionally kill an animal, other than: for food while hunting, or to help the animal such as through euthanasia
No water	An animal left without clean water for 24 h or more

**Table 2:** Belief statements used to assess attitudes towards reporting mistreatment including behavioural beliefs (BB), subjective norms (SN), perceived behavioural control (PBC), and other (ATT).

Belief Statement	1	2	3	4	5
BB1. To what extent do you think reporting animal mistreatment would be likely to help the animal?	Very unlikely	Unlikely	Neither likely or unlikely	Likely	Very Likely
BB2. Reporting animal mistreatment is the right thing to do	Strongly disagree	Disagree	Neither agree nor disagree	Agree	Strongly agree
BB3. Reporting animal mistreatment is not my responsibility	Strongly disagree	Disagree	Neither agree nor disagree	Agree	Strongly agree
SN1. People whose opinions matter to me, like friends or family, would expect me to make a report if I witnessed animal mistreatment	Strongly disagree	Disagree	Neither agree nor disagree	Agree	Strongly agree
SN2. My friends would think it was none of my business or criticise me if I reported animal mistreatment	Strongly disagree	Disagree	Neither agree nor disagree	Agree	Strongly agree
PBC1. I don’t have time to report animal mistreatment	Strongly disagree	Disagree	Neither agree nor disagree	Agree	Strongly agree
PBC2. How confident would you be in recognising the signs of animal mistreatment	Not at all confident	Not very confident	Reasonably confident	Mostly confident	Fully confident
PBC3. How confident would you be in knowing what to do if you witnessed animal mistreatment	Not at all confident	Not very confident	Reasonably confident	Mostly confident	Fully confident
PBC4. How confident would you be in knowing who to report animal mistreatment to	Not at all confident	Not very confident	Reasonably confident	Mostly confident	Fully confident
ATT1. I would be unlikely to report animal mistreatment unless it was affecting me	Strongly disagree	Disagree	Neither agree nor disagree	Agree	Strongly agree
ATT2. I would be concerned about negative repercussions for me if I reported animal mistreatment	Strongly disagree	Disagree	Neither agree nor disagree	Agree	Strongly agree

The draft questionnaire was reviewed by colleagues at the Animal Welfare Science Centre, University of Melbourne; RSPCA Victoria; and Social Research Centre, Australian National University, with feedback incorporated in an iterative process. Questions were pre-tested for comprehension and relevance with a range of people known to the researchers. The first evening of survey delivery was used as a pilot, with feedback from telephone operators leading to minor grammatical modifications.

**Table 3:** High-ranked and low-ranked Local Government Areas (LGA) were paired based on population, Social Economic Index For Areas (SEIFA), and Local Government Comparator Group (LGCG).

LGCG	Pair 1		Pair 2		Pair 3	
	Regional City		Metropolitan		Interface	
**RSPCA Case Data Ranking**	High	Low	High	Low	High	Low
**LGA**	Latrobe	Mildura	Melbourne	Stonnington	Yarra Ranges	Wyndham
Population	73,257	53,878	135,959	103,832	149,537	199,715
SEIFA [29]	3	5	72	78	57	54
# cruelty reports 2017–2018	259	50	262	79	282	266
# cruelty reports 2013–2018	1175	168	1438	323	1583	1124
Average # reports per year	235	33.6	287.6	64.6	316.6	224.8
Average per year per 10,000 people	32.08	6.24	21.15	6.22	21.17	11.26

**Table 4:** No difference between paired high ranked (HR) and low ranked (LR) Local Government Areas in the proportion of people who had witnessed at least one incident of mistreatment in the past 12 months.

Region Type	Rank	LGA	Count No	Count Yes	Proportion Yes	g	F	Interpretation
Regional	HR	Latrobe	202	98	0.327	0	0.093	No difference
	LR	Mildura	210	91	0.302			
Metro	HR	Melbourne	228	72	0.240	0	0.002	No difference
	LR	Stonnington	229	71	0.237			
Interface	HR	Yarra Ranges	225	75	0.250	0	0.699	No difference
	LR	Wyndham	245	55	0.183			

Decision-based critical F value for F[0.05]; df_1_:5,df_2_:infinity = 1.372.

**Table 5:** Sum of action responses by participants who had witnessed mistreatment across each Local Government Area and overall.

Action	Latrobe	Mildura	Melbourne	Stonnington	Yarra Ranges	Wyndham	Overall
1. Reported to RSPCA Vic	37	7	17	0	8	18	87
2. Reported to Council	31	48	9	10	19	13	130
3. Reported to Government Department	5	5	5	0	3	3	21
4. Reported to Police	13	15	3	0	4	4	39
5. Discussed with a professional	19	20	5	5	12	15	76
6. Discussed with family or friend	33	23	24	7	12	23	122
7. Other (please specify)	7	10	8	1	2	5	33
8. Nothing	48	55	44	52	39	18	256
9. Approached person directly	30	24	19	18	34	12	137
10. Helped animal directly	14	15	14	5	6	11	65
11. Don’t know	0	1	0	0	0	0	1
**Total responses**	237	223	148	98	139	122	967

**Table 6:** Significant differences in the propensity to report to RSPCA Victoria between High Ranked (HR) and Low Ranked (LR) Local Government Areas in Regional and Metro Regions.

Region Type	Rank	LGA	Count Reported to RSPCA	Count all Actions Taken	Proportion of all Actions Taken	g	F	Interpretation
Regional	HR	Latrobe	37	237	0.156	0.308	4.366	Sig. difference
	LR	Mildura	7	223	0.031			
Metro	HR	Melbourne	17	148	0.115	0.284	1.900	Sig. difference
	LR	Stonnington	0	98	0			
Interface	HR	Yarra Ranges	8	139	0.058	0	1.285	No difference
	LR	Wyndham	18	122	0.148			

Decision-based critical F value for F[0.05]; df_1_:5, df_2_:infinity= 1.372.

**Table 7:** Biserial correlations between attitudes items and whether a person who witnessed mistreatment reported it or not were small.

Attitude Item	r	Sig (2-Tailed)	*n*
BB1. To what extent do you think reporting animal mistreatment would be likely to help the animal?	0.029	0.540	456
BB2. Reporting animal mistreatment is the right thing to do	0.020	0.669	461
BB3. Reporting animal mistreatment is not my responsibility	−0.076	0.105	459
SN1. People whose opinions matter to me, like friends or family, would expect me to make a report if I witnessed animal mistreatment:	0.097 *	0.037	460
SN2. My friends would think it was none of my business or criticise me if I reported animal mistreatment	−0.105 *	0.026	454
PBC1. I don’t have time to report animal mistreatment	−0.025	0.589	460
PBC2. How confident would you be in recognising the signs of animal mistreatment	0.086	0.065	462
PBC3. How confident would you be in knowing what to do if you witnessed animal mistreatment	0.125 **	0.007	461
PBC4. How confident would you be in knowing who to report animal mistreatment to	0.157 **	0.001	460
ATT1. I would be unlikely to report animal mistreatment unless it was affecting me	0-.051	0.276	457
ATT2. I would be concerned about negative repercussions for me if I reported animal mistreatment	0-.017	0.723	457

* *p* < 0.05; ** *p* < 0.01.

**Table 8:** Associations between demographic variables and reporting behaviour.

Variable	Pearson Chi-square Value	df	*p*-Value	Interpretation
Gender	1.599	1	0.206	Not sig
Age group	3.894	7	0.792	Not sig
Country of birth	4.776	11	0.942	Not sig.
Education	5.838	7	0.559	Not sig.
Income	16.825	13	0.207	Not sig.
Animal ownership	.203	1	0.478	Not sig.
LGA Type	10.746	2	0.005	Significant
LGA	12.189	5	0.032	Significant
